# Drug Repositioning Based on Single-Cell Transcriptomics Data Identifies Quinidine as a Potential Drug for Treating Osteoporosis

**DOI:** 10.3390/genes17091060

**Published:** 2026-08-31

**Authors:** Rong Wang, Jia-Li Li, Kun Liu, Xuan Lu, Qian-Qian Zhang, Qin Zeng, Yun Deng, Xiao-Chao Qu, Xiang-Ding Chen, Li-Jun Tan

**Affiliations:** 1 Laboratory of Molecular and Statistical Genetics, College of Life Sciences, Hunan Normal University, Changsha 410081, China; 202420142860@hunnu.edu.cn (R.W.);; 2 Zebrafish Genetics Laboratory, College of Life Sciences, Hunan Normal University, Changsha 410081, China

**Keywords:** osteoporosis, drug repositioning, single-cell RNA sequencing, quinidine, mendelian randomization, zebrafish

## Abstract

Osteoporosis (OP) is characterized by low bone mass and impaired bone microarchitecture, and the long-term use of current therapies is limited by adverse effects and poor adherence. This study sought to identify drugs that could be repurposed for glucocorticoid-induced osteoporosis. Methods: Single-cell RNA-sequencing data from two subjects with osteoporotic fractures and three control subjects were analyzed to identify transcriptional changes in osteoblasts. Candidate drugs were screened using ASGARD and L1000 drug-response signatures. Two-sample Mendelian randomization was used to examine associations between genetically predicted expression of drug-target genes and osteoporosis risk. Quinidine was tested in dexamethasone-treated Tu and Tg(*Ola.Sp7*:nlsGFP) zebrafish larvae using Alizarin Red staining, fluorescence imaging, and qRT-PCR. Adult zebrafish were assessed by micro-CT. Results: Four candidate drugs were identified, of which quinidine was selected for experimental evaluation. Genetically predicted SCN5A expression was positively associated with osteoporosis risk. Quinidine partially restored cranial mineralization in dexamethasone-treated larvae, with the greatest effect at 10 μmol/L; the 100 μmol/L treatment was less effective. Quinidine also altered the expression of several bone-remodeling-related genes. In adult zebrafish, quinidine treatment was associated with higher bone mineral density and bone volume fraction than dexamethasone treatment alone. Conclusions: Quinidine alleviated several dexamethasone-induced skeletal phenotypes in zebrafish and represents a candidate for further drug-repurposing studies in osteoporosis. Direct validation of its molecular mechanism and safety profile is still required.

## 1. Introduction

Osteoporosis (OP) is a systemic skeletal disease characterized by reduced bone mass and deterioration of bone tissue microstructure, leading to decreased bone strength and stability. Its most serious consequence, fracture, can result in patient disability or even death [1]. With the acceleration of global population aging, the incidence and prevalence of OP are increasing year by year. It is estimated that by 2050, osteoporotic fractures in China will rise to 5.91 million cases, and the treatment cost will reach $27.48 billion [2], posing a huge economic and medical burden.

The pathogenesis of osteoporosis is complex, primarily involving an imbalance in bone remodeling, changes in endocrine hormones, and dysregulation of the immune microenvironment [3,4,5]. Bone remodeling is a dynamic balance between bone formation and bone resorption. In osteoporosis, a net decrease in bone mass occurs when osteoclast-mediated bone resorption exceeds osteoblast-mediated bone formation [6]. Multiple signaling pathways are involved in the regulation of bone metabolism, including the Wnt/β-catenin signaling pathway, the *RANKL*/*RANK*/*OPG* signaling pathway, and the *NF-κB* signaling pathway, among others [7,8]. Changes in endocrine hormone levels (such as the sharp decline in estrogen secretion after menopause and the long-term use of glucocorticoids) and disturbances in the immune microenvironment can both lead to osteoporosis by affecting bone remodeling [9,10].

Therapeutic agents of osteoporosis are broadly classified into anti-resorptive and bone-forming drugs. Anti-resorptive drugs mainly include bisphosphonates, estrogens, calcitonin, and RANKL inhibitors. Bone-forming drugs, such as the parathyroid hormone analogue Teriparatide, promote the proliferation, differentiation, and activity of osteoblasts [11]. Although these drugs can, to a certain extent, slow down bone resorption or promote bone formation, long-term use presents certain limitations and side effects. For example, prolonged use of bisphosphonates can lead to osteonecrosis of the jaw [12] and atypical femoral fractures [13], and long-term use of bone-forming drugs may also cause severe adverse events such as nausea, dizziness, and leg cramps [14]. Therefore, the discovery of new drug targets and safe, effective therapeutic strategies is an urgent necessity.

Drug repositioning (or drug repurposing) is the process of redeveloping drugs already approved for treating one disease to treat other diseases. Compared to traditional new drug development, which requires 9–12 years and an approximate cost of $1.24 billion [15,16], drug repositioning can significantly reduce both time and cost. The computational pipeline for drug repositioning based on single-cell data, ASGARD [17], offers a unique advantage. This method considers the heterogeneity of all cell clusters and inputs differentially expressed genes into the L1000 drug response dataset to identify drugs that can significantly reverse the gene expression profile. Compared to methods developed by Alakwaa et al. [18] and Wang Z et al. [19], ASGARD has demonstrated higher AUC values across multiple datasets, indicating superior reliability and robustness.

The zebrafish is an ideal model for osteoporosis research due to its strong developmental capacity, low rearing cost, and short experimental cycle. Zebrafish share approximately 70% gene homology with humans [20], and their skeletal development and molecular regulation mechanisms are similar to those in humans. Dexamethasone is a commonly used drug for inducing an osteoporosis model in zebrafish [21].

This study aims to screen candidate drugs for osteoporosis treatment using a computational pipeline for drug repositioning based on single-cell transcriptomics data. Subsequently, Mendelian Randomization analysis will be used to infer the causal relationship between the expression of drug target gene and osteoporosis. Finally, experimental validation will be performed using a dexamethasone-induced osteoporosis zebrafish model. This comprehensive approach, spanning from computational prediction to in vivo validation, will evaluate the potential of candidate drug in the prevention and treatment of osteoporosis.

## 2. Materials and Methods

### 2.1. Acquisition and Analysis of Single-Cell Transcriptomics Data for Osteoporosis

Single-cell transcriptomics dataset for non-union fractures (GSE217792) [22] was retrieved from the Gene Expression Omnibus (GEO) database. Two osteoporotic fracture samples (GSM6726765 and GSM6726769) were selected as the experimental group, and three healthy samples (GSM6726762, GSM6726763, and GSM6726764) were selected as the control group.

The single-cell dataset was subjected to quality control and normalization using the Seurat R package. Cells with fewer than 200 or more than 6000 detected genes, and those with a mitochondrial gene proportion exceeding 10%, were filtered out to remove low-quality cells and technical noise.

The ElbowPlot function was used to observe the variance contribution rate of the principal components. The top 42 principal components were selected for dimensionality reduction. Cell clustering was performed with a resolution of 0.6, and visualization was achieved using UMAP. Differentially Expressed Genes (DEGs) were identified using the FindAllMarkers function (Logfc.threshold = 0.25). Cell clusters were annotated by integrating results with existing literature and the CellMarker database (http://bio-bigdata.hrbmu.edu.cn/CellMarker/ accessed on 27 May 2026).

Sample integration and batch-effect correction were explored using Harmony (grouped by Sample_ID) and Seurat IntegrateLayers. The primary clustering (resolution = 0.6) and subsequent differential expression analyses were performed on the quality-controlled dataset. Formal doublet detection/removal and ambient RNA correction were not applied. Donor contribution across clusters was examined to assess whether any major cell type was disproportionately contributed by a single donor (Supplementary Figure S1).

To address the limited number of biological replicates (*n* = 2 osteoporotic fracture donors versus n = 3 control donors) and potential pseudoreplication, we additionally performed donor-level pseudobulk differential expression analysis of osteoblasts, which identified five genes meeting the predefined criteria of |log_2_FC| > 1 and adjusted *p* < 0.05, namely, APOL4, CD36, ACAN, IFITM1, and AL109811.3 (Supplementary Table S1). Given the limited number of biological donors, these results were interpreted as exploratory.

Gene Ontology (GO) functional enrichment analysis was performed on the DEGs of the osteoblast cluster (|LogFC| > 1, adjusted *p*-value < 0.05) to investigate their biological processes and molecular functions.

### 2.2. Drug Repositioning Analysis Based on Single-Cell Transcriptomics Data

Candidate drugs were screened using the computational pipeline for drug repositioning based on single-cell transcriptomics data (ASGARD) [17]. The disease group and the healthy group were paired for analysis to identify DEGs (*p* < 0.05, |LogFC| > 1). The set of osteoblast DEGs was then compared against the L1000 drug response dataset to identify drugs capable of significantly reversing the differential gene expression profile. The screened potential candidate drugs were ranked according to a defined drug score formula. The drug score formula is as follows:

$$\mathrm{Drug}\;\mathrm{score}=\sum_{k=1}^{n}\left(\frac{\mathrm{Num}{\left(Cell\right)}_{k}}{\mathrm{Num}(Total.Cell)}*(-\log_{10}^{FDR_{k}})*\frac{\mathrm{Num}{\left(ReversedGene\right)}_{k}}{\mathrm{Num}{\left(DiseasedGene\right)}_{k}}\right)$$

where *k* represents a specific single-cell cluster and *n* represents all disease-associated single-cell clusters. The raw drug therapeutic score reported in Table 1 has no fixed upper bound, as it is calculated by summing weighted contributions across all disease-associated cell clusters. For cross-sample comparability, a standardized drug score bounded between 0 and 1 was additionally calculated based on the rank of each drug, with the formula as follows:

$$\mathrm{Standardized}\;\mathrm{Drug}\;\mathrm{Score}=1-\frac{\mathrm{Rank}(Drug)}{\mathrm{Total}\;\mathrm{Num}(Drug)}$$

### 2.3. Mendelian Randomization Analysis

To infer the causal relationship between the expression of candidate drug target gene and osteoporosis, Mendelian Randomization analysis was performed. The target genes of the candidate drug were queried on the DrugBank website (https://go.drugbank.com/; accessed on 27 May 2026). The Ensembl sequence numbers for the target genes were retrieved from the NCBI website (https://www.ncbi.nlm.nih.gov/; accessed on 27 May 2026). Expression Quantitative Trait Loci (eQTLs) for the target genes were then obtained from the IEU OpenGWAS database (https://gwas.mrcieu.ac.uk/; accessed on 27 May 2026). The eQTLs for SCN5A (OpenGWAS ID: eqtl-a-ENSG00000183873) were obtained from the eQTLGen Consortium [23] (n = 26,022) and are derived from whole blood. The criteria for selecting instrumental variables were: a *p*-value threshold of *p* < 5 × 10^−6^ for genome-wide significance, an r^2^ threshold of 0.1, and a physical distance threshold greater than 10000 kb. This ensured the selection of independent SNPs to serve as instrumental variables. The Genome-Wide Association Study (GWAS) data for osteoporosis was obtained from the IEU OpenGWAS database, comprising 455,386 controls and 7547 osteoporosis cases.

The analysis was performed using the TwoSampleMR R package [24]. Three primary methods were employed: Inverse Variance Weighted (IVW), MR-Egger, and Weighted Median (WM). The IVW method was used as the main approach to assess the causal relationship between the drug target and osteoporosis, with the other two methods serving as robustness checks.

Cochran’s Q test [25] was used to assess heterogeneity, and the MR-Egger intercept test [26] was employed to evaluate pleiotropy. A *p*-value greater than 0.05 indicates the absence of significant heterogeneity or pleiotropy, while a *p*-value less than 0.05 indicates the presence of significant heterogeneity or pleiotropy. Instrument strength was assessed using the F-statistic, calculated as (β/SE)^2^ for each SNP (Supplementary Table S2). Leave-one-out analysis was performed to evaluate the influence of individual SNPs on the overall estimate (Supplementary Figure S2).

### 2.4. Zebrafish Animal Model Experimental Validation

To validate the osteogenic effect of candidate drug, we used a dexamethasone-induced osteoporosis zebrafish model. Tu wild-type zebrafish and Tg(*Ola.Sp7*:nlsGFP) transgenic zebrafish were used. The zebrafish were maintained at 28 °C, with the pH kept between 7 and 8, and a light-dark cycle of 14 h light/10 h dark. The zebrafish were fed with brine shrimp three times a day. Embryos were collected in E3 medium and maintained at 28 °C. The E3 medium was replaced twice daily, and dead embryos were removed during routine monitoring. On the third day after fertilization, larvae with normal morphology were selected for subsequent drug treatment experiments. No additional environmental enrichment was provided during the experimental period. The transgenic zebrafish Tg(*Ola.Sp7*:nlsGFP) express green fluorescent protein (nlsGFP) in their osteoblasts, allowing for observation of osteoblast activity and bone mineralization.

Three-day-old wild-type Tu and Tg(*Ola.Sp7*:nlsGFP) larvae with normal morphology were selected and allocated to the experimental groups. The sample size (*n* = 15–20 per group) was chosen according to previously reported protocols using dexamethasone-induced zebrafish osteoporosis models and a priori power analysis was not performed. The experimental design included a control group treated with 0.2% DMSO (Beijing Coolaber Science & Technology Co., Ltd., Beijing, China) and a model group treated with 10 μmol/L dexamethasone (MedChemExpress, Monmouth Junction, NJ, USA). Additionally, three combination treatment groups were established, consisting of 10 μmol/L dexamethasone plus quinidine (MedChemExpress, Monmouth Junction, NJ, USA) at concentrations of 1, 10, and 100 μmol/L, as well as three separate quinidine treatment groups at the same concentrations. Twenty larvae were initially assigned to each treatment group in a six-well plate. The larvae were treated continuously for 5 days, and the treatment solutions were replaced every 24 h. After treatment, 15 larvae from each group were randomly selected for Alizarin Red staining or fluorescence imaging and were quantified individually. Thus, 20 refers to the number of larvae initially assigned to each treatment group, whereas *n* = 15 refers to the number of larvae included in the imaging-based quantitative analysis. Each quantified larva contributed one measurement to the analysis.

Tu wild-type zebrafish were stained with Alizarin Red (Beijing Solarbio Science & Technology Co., Ltd., Beijing, China). Images were captured using a stereo microscope (Leica Microsystems GmbH, Wetzlar, Germany), and the ventral side of the zebrafish larvae was observed. Bone mineralization area and grayscale values were quantified using Image J software (Version 1.52a; National Institutes of Health, Bethesda, MD, USA). Image quantification was performed by an investigator blinded to group allocation. Tg(*Ola.Sp7*:nlsGFP) transgenic zebrafish were subjected to fluorescent stereo microscopy imaging. The fluorescent area and fluorescent intensity of the skull were quantitatively analyzed, which represents the degree of bone mineralization. For larval imaging analyses, the experimental unit was an individual larva. For qRT-PCR analysis, each pooled sample containing approximately 50–60 larvae constituted one biological replicate and was treated as one experimental unit. For adult Micro-CT analysis, the experimental unit was an individual zebrafish.

The principal larval imaging outcomes were cranial mineralization area and integrated density measured by Alizarin Red staining, together with cranial fluorescent area and fluorescence intensity measured in Tg(*Ola.Sp7*:nlsGFP) larvae. Secondary outcomes included the expression levels of bone-associated genes measured by qRT-PCR, bone mineral density (BMD) and bone volume fraction (BV/TV) measured by Micro-CT in adult zebrafish, mortality, gross morphological abnormalities, and abnormal swimming behavior. No single primary outcome was prespecified for sample-size determination because no a priori power calculation was performed.

Whole larvae were collected for qRT-PCR analysis. For each biological replicate, approximately 50–60 whole larvae from the same treatment group were pooled for total RNA extraction using TransZol Up reagent (TransGen Biotech Co., Ltd., Beijing, China). Three independent biological replicates were prepared for each group, and cDNA was synthesized using a TransScript Uni All-in-One reverse-transcription kit (TransGen Biotech Co., Ltd., Beijing, China). Quantitative real-time PCR (qRT-PCR) was performed using F488 SYBR qPCR Mix (Universal; BestEnzymes Biotech Co., Ltd., Lianyungang, China) on a Bio-Rad real-time PCR system (Bio-Rad Laboratories, Inc., Hercules, CA, USA) to measure the expression levels of osteoblast marker genes (runx2b, col2a1a, and sp7) and osteoclast-related genes (spp1, mmp13, ctsk, and opg). β-actin was used as the internal reference gene. To verify the effect of quinidine on mature bone tissue, an adult zebrafish bone mineral density (BMD) detection experiment was established. Adult zebrafish aged 3–5 months were randomly assigned to three groups, with five fish per group: the control group, the dexamethasone model group, and the dexamethasone plus quinidine treatment group. The dexamethasone model group was exposed to 10 μmol/L dexamethasone for 14 days, whereas the dexamethasone plus quinidine treatment group received 10 μmol/L dexamethasone together with 10 μmol/L quinidine during the same treatment period. The 14-day treatment duration was predetermined based on established durations used in comparable glucocorticoid-induced osteoporosis models prior to initiating the experiment. No mortality was observed in any treatment group during the 14-day experimental period. Fish in the dexamethasone plus quinidine treatment group exhibited noticeably sluggish swimming behavior and altered swimming patterns compared with the control and dexamethasone-only groups, consistent with the known pharmacological effects of quinidine on cardiac and neuromuscular ion channels. After the experiment, Micro-CT analysis was performed to evaluate the effect of quinidine on the BMD and structure of the adult zebrafish. A total of *n* = 5 adult zebrafish per group (mixed sex) were used for Micro-CT analysis. Fish were randomly assigned to groups, and Micro-CT scanning and BMD/BV-TV quantification were performed by an investigator blinded to group identity. No mortality or overt toxicity was observed during the 14-day treatment period in any group.

### 2.5. Statistical Analysis

Statistical analysis was performed using GraphPad Prism Version 9.0.0 (GraphPad Software, San Diego, CA, USA). The unpaired *t*-test was used for comparisons between two groups, and one-way analysis of variance (ANOVA) was used for comparisons among multiple groups. For one-way ANOVA, the Holm–Šídák method was applied for multiple pairwise comparisons. Given the multiple concentration groups, genes, and endpoints examined, findings should be interpreted with awareness of the increased risk of Type I error from multiple comparisons. All data are presented as mean ± standard deviation (SD), and *p*-value of < 0.05 was considered statistically significant. For larval Alizarin Red staining and fluorescence quantification, 15 larvae were ran-domly selected from the 20 initially assigned to each group. For qRT-PCR, three independent biological replicates were performed, each using RNA pooled from approximately 50–60 larvae. For adult Micro-CT, *n* = 5 fish per group were analyzed.

## 3. Result

### 3.1. Single-Cell Transcriptomic Landscape of Osteoporotic Fractures

**Figure 1:**
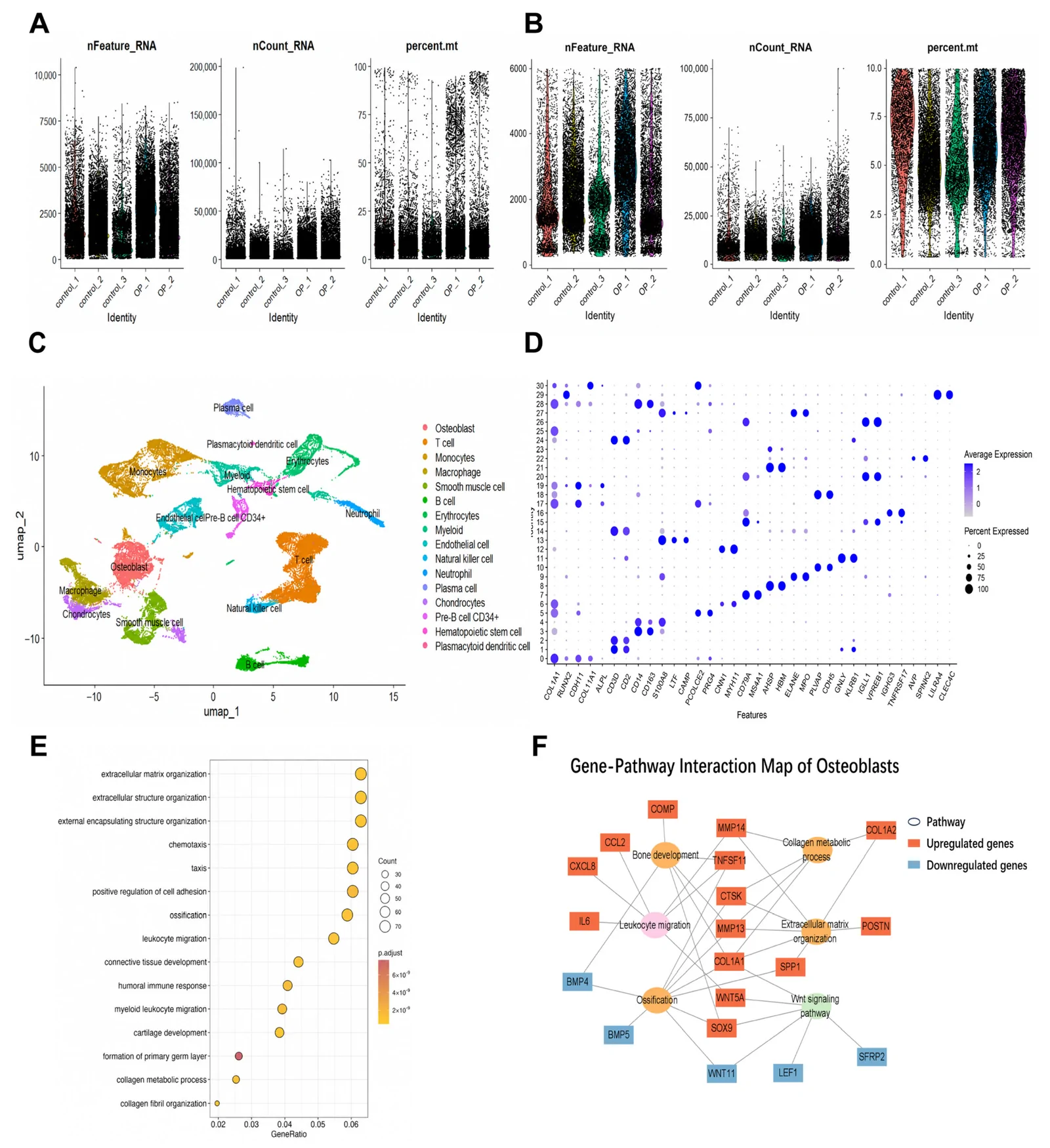
Single-cell transcriptomic landscape of osteoporotic fractures and identification of Quinidine as a candidate drug. (**A**) The contents of nFeature_RNA, nCount_RNA, and percent.mt before the quality control of single-cell transcriptome data of osteoporosis. (**B**) The contents of nFeature_RNA, nCount_RNA, and percent.mt after the quality control of single-cell transcriptome data of osteoporosis. In (**A**,**B**), colors distinguish individual samples: control_1–3 represent control subjects, whereas OP_1–2 represent osteoporotic fracture subjects. (**C**) The expression profiles of marker genes across all identified cell clusters. (**D**) UMAP plot of the annotation results of single-cell transcriptome data of osteoporosis. (**E**) Functional enrichment analysis of differentially expressed genes in the osteoblast cluster. (**F**) Gene–Pathway Interaction Map of Osteoblasts. The network details the specific linkages between enriched GO terms and their driver genes.

For single-cell transcriptomic analysis, three control subjects and two osteoporotic fracture subjects were selected in this study (Figure 1A). After quality control, a total of 29,742 cells were analyzed, comprising 14,410 cells from control group and 15,332 cells from osteoporotic fracture group (Figure 1B). Using the criteria of PC = 42 and a resolution of 0.6, cell clustering was performed, resulting in the identification of 31 distinct cell clusters.

Based on the DEGs of each cell cluster, combined with evidence from published literature [22,27] and the CellMarker database, we systematically identified specific marker genes for individual cell clusters (Figure 1C). The 31 cell clusters were annotated into 16 distinct cell types: Osteoblasts, Chondrocytes, T cells, Monocytes, Neutrophils, Macrophages, Smooth muscle cells, B cells, Erythrocytes, Myeloid cells, Endothelial cells, Natural killer cells, pre-B cell CD34+, Plasma cells, Hematopoietic stem cells, and Plasmacytoid dendritic cells (Figure 1D).

A total of 1647 DEGs were identified between the osteoblast cluster of the osteoporotic fracture group and the control group (|LogFC| > 1 and adjusted *p*-value < 0.05). When the same comparison was re-evaluated at the donor level using pseudobulk analysis (DESeq2), only a small number of genes remained significant after multiple-testing correction (Supplementary Table S1), indicating that the original single-cell differential expression results were partly influenced by treating individual cells as independent observations. Gene Ontology analysis showed the DEGs were primarily enriched in the following biological processes: extracellular matrix organization, chemotaxis, ossification, connective tissue development, myeloid leukocyte migration, and cartilage development (Figure 1E). The enriched molecular functions included collagen metabolic process and collagen fibril organization. We also constructed a gene–pathway interaction network (Figure 1F). This network revealed that upregulated genes (including COL1A1, SPP1, and MMP13) were closely associated with extracellular matrix organization and Wnt signaling, while downregulated genes such as BMP4 and LEF1 were simultaneously enriched in opposing functional cascades. These findings collectively delineate the core molecular dysregulation underlying osteoporosis pathogenesis.

### 3.2. Identification of Quinidine as a Potential Therapeutic Agent via Drug Repositioning and Mendelian Randomization

Using the ASGARD computational pipeline for drug repositioning on the differentially expressed genes of the osteoblast cluster, four potential candidate drugs for osteoporosis treatment were screened: perphenazine, imatinib, quinidine, and vinblastine (Table 1). To prioritize among these candidates, we applied the following pre-defined selection criteria: (i) statistically significant drug score with FDR < 0.05; (ii) primary action on the osteoblast cluster (the cell type of primary interest in this study); (iii) clinical accessibility (approved drug with established safety profile); and (iv) limited prior investigation specifically in the context of osteoporosis or bone metabolism, so that the candidate would represent a novel repurposing opportunity. Based on these criteria, perphenazine and imatinib were deprioritized. Previous studies have shown that perphenazine is associated with reduced bone mineral density and increased incidence of osteoporosis, while imatinib can decrease osteoclast number and activity, leading to bone metabolism disorders. These reported effects on bone are either detrimental or complex, making them less suitable as osteoprotective candidates. Vinblastine has also been linked to interference with calcium homeostasis and osteoblast function. In contrast, quinidine (a Class Ia antiarrhythmic sodium-channel blocker) has not previously been investigated for osteoporosis, satisfied all selection criteria, and ranked third by drug score. Therefore, quinidine was selected for subsequent Mendelian randomization analysis and experimental validation.

Quinidine has seven drug targets: *SCN5A*, KCNK1, KCNK6, KCNH2, ADRA1A, ADRA1B, and ADRA1D. Among these, *SCN5A*, KCNK6, and KCNH2 have corresponding eQTLs available in the IEU OpenGWAS database. For the *SCN5A* drug target, four SNPs (rs6810361, rs61558743, rs11720524 and rs7355975) were selected as instrumental variables.

**Figure 2:**
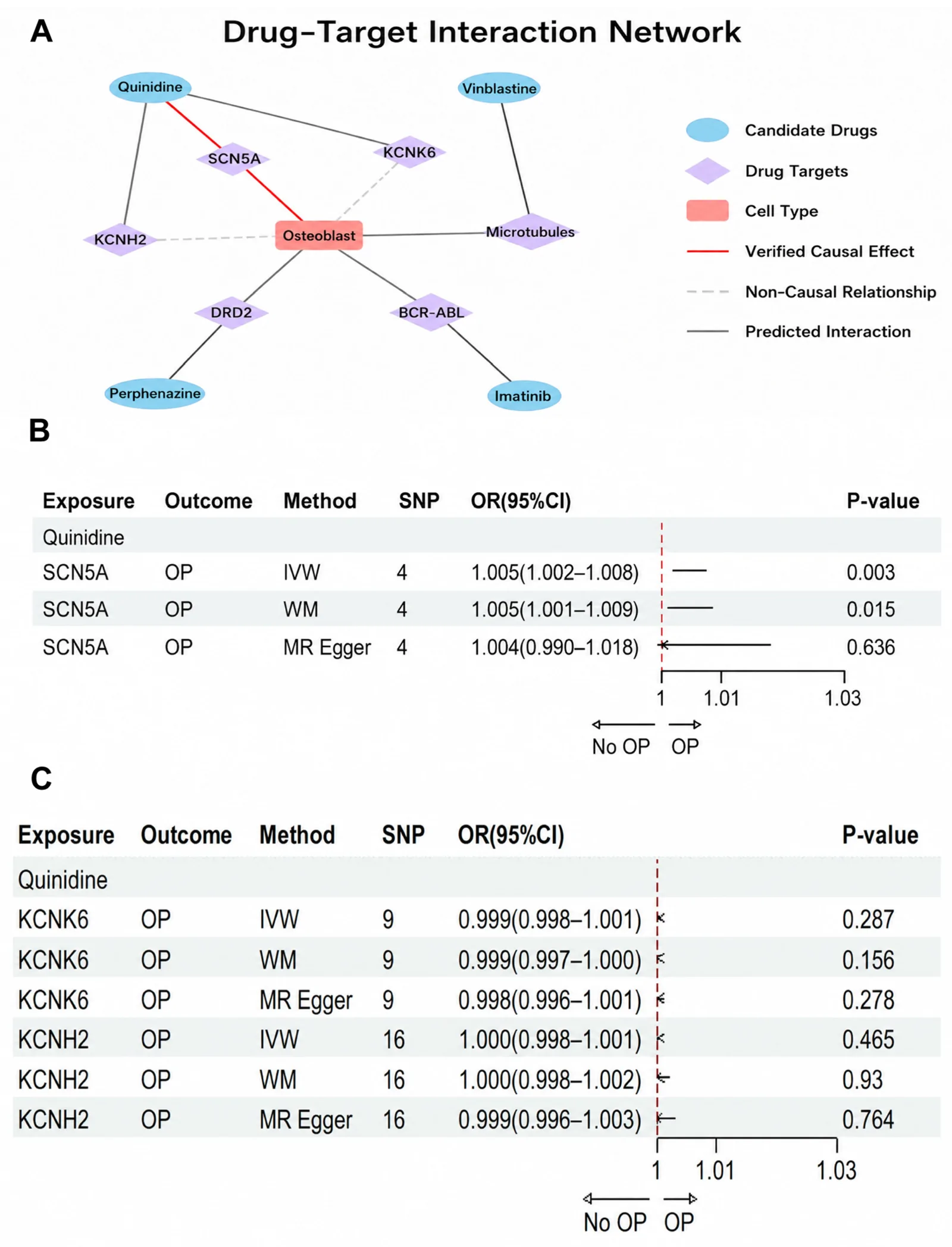
Drug–target interaction network and Mendelian randomization analysis. (**A**) Drug–target interaction network. The red path highlights SCN5A as a primary target of quinidine. (**B**) Association between genetically predicted SCN5A expression and osteoporosis risk. (**C**) Association between genetically predicted expression of KCNK6/KCNH2 and osteoporosis risk.

To further prioritize the candidate drugs, we first constructed a drug–target interaction network (Figure 2A), which identified SCN5A as a primary target of quinidine. Two-sample MR analysis using the inverse-variance weighted (IVW) method showed that genetically predicted SCN5A expression was positively associated with osteoporosis risk (OR = 1.005, 95% CI: 1.002–1.008, *p* = 0.003; Figure 2B). Directionally consistent estimates were obtained using the weighted median method (OR = 1.005, 95% CI: 1.001–1.009, *p* = 0.015); the MR-Egger estimate was directionally consistent but did not reach statistical significance (OR = 1.004, 95% CI: 0.990–1.018, *p* = 0.636), which may reflect limited statistical power given the small number of instrumental variables (n = 4). No evidence of heterogeneity (Cochran’s Q test, *p* = 0.732 for IVW and *p* = 0.530 for MR-Egger) or horizontal pleiotropy (MR-Egger intercept = 9.95 × 10^−5^, *p* = 0.899) was detected. F-statistics for the four instrumental variables ranged from 21.5 to 95.5 (mean F = 42.0), indicating no evidence of weak instrument bias. Leave-one-out analysis confirmed that the overall causal estimate was not driven by any single SNP, as the 95% confidence interval excluded the null across all leave-one-out iterations. No significant association was found for KCNK6 or KCNH2 (Figure 2C). It should be noted that the MR analysis evaluates the association between genetically predicted SCN5A expression levels and osteoporosis risk; it does not directly test the therapeutic effect of quinidine. Quinidine acts primarily by blocking NaV1.5 channel activity (encoded by SCN5A) rather than by suppressing SCN5A gene expression, and it also has additional ion-channel and adrenergic targets. Therefore, the observed genetic association is consistent with, but does not by itself establish, a protective effect of quinidine on bone. Given the small effect size and the indirect nature of the inference, the MR result should be interpreted as hypothesis-generating. The subsequent zebrafish experiments evaluated the preliminary osteoprotective activity of quinidine but did not directly validate an SCN5A-dependent mechanism.

### 3.3. Quinidine Ameliorates Dexamethasone-Induced Osteoporosis

**Figure 3:**
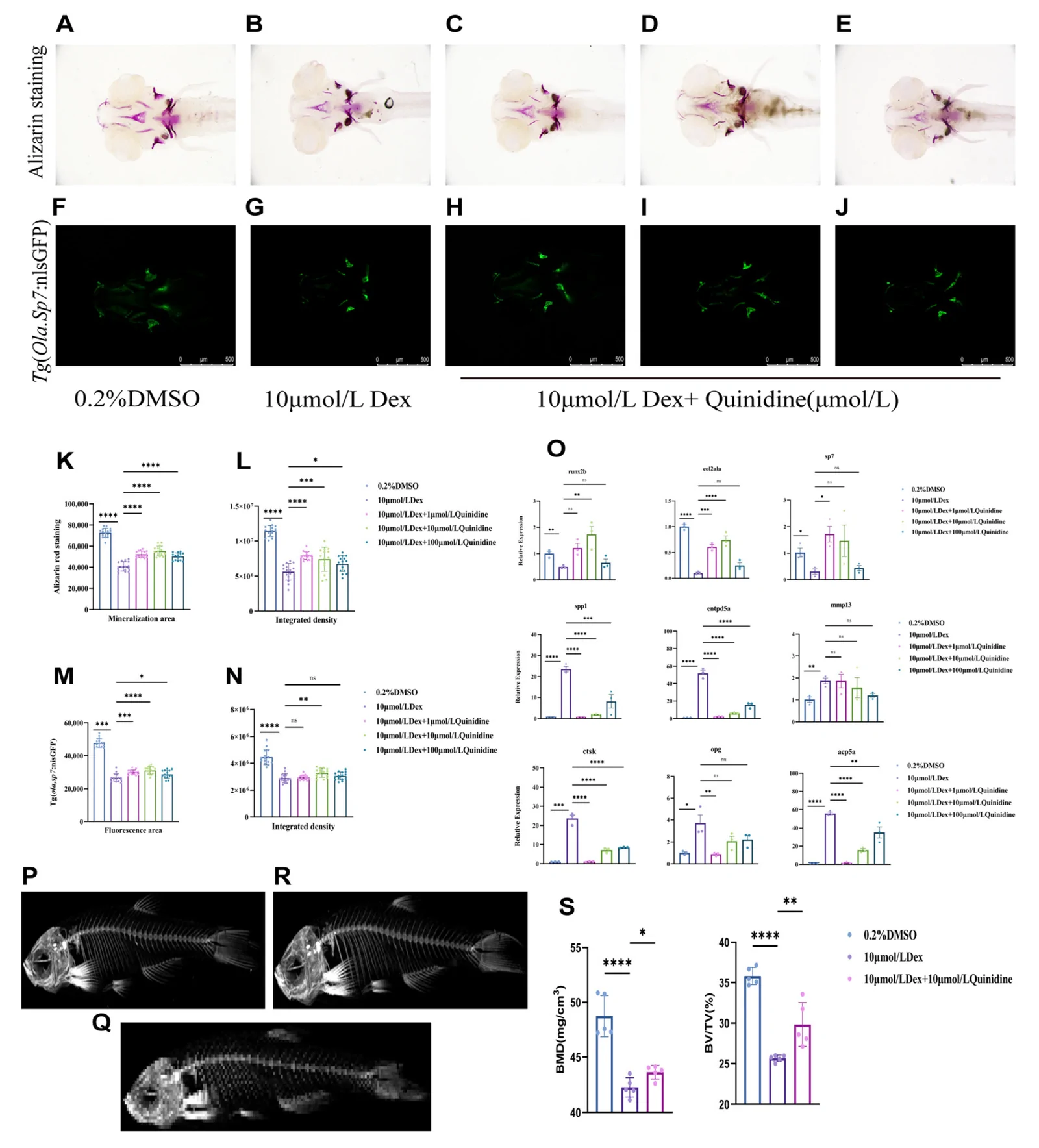
Quinidine ameliorates Dexamethasone-induced osteoporosis in a non-monotonic, concentration-dependent manner, with the most pronounced effect observed at 10 μmol/L. (**A**–**J**) Representative images of Alizarin Red staining of Tu zebrafish and fluorescence of *Tg*(*Ola.Sp7*:nlsGFP) after quinidine bath treatment. (**A**,**F**) 0.2% DMSO group; (**B**,**G**) 10 μmol/L Dex group; (**C**,**H**) 10 μmol/L Dex + 1 μmol/L quinidine group; (**D**,**I**) 10 μmol/L Dex + 10 μmol/L quinidine group; (**E**,**J**) 10 μmol/L Dex + 100 μmol/L quinidine group. (**K**–**N**) Quantitative results of the mineralized area and intensity of Alizarin Red staining and fluorescence after quinidine bath treatment. (**K**) Quantitative results of the mineralized area of Alizarin Red staining in Tu zebrafish; (**L**) Quantitative results of the mineralized intensity of Alizarin Red staining in Tu zebrafish; (**M**) Quantitative results of the fluorescent mineralized area of *Tg*(*Ola.Sp7*:nlsGFP) transgenic zebrafish; (**N**) Quantitative results of the fluorescent mineralized intensity of *Tg*(*Ola.Sp7*:nlsGFP) transgenic zebrafish. (**O**) Results of qRT-PCR for marker genes of osteoblasts and osteoclasts after quinidine bath treatment. (**P**–**R**) Representative three-dimensional micro-CT images of vertebrae showing bone density in the control (**P**), model ((**Q**), 10 µmol/L Dex), and quinidine-treated groups ((**R**), 10 µmol/L Dex + 10 µmol/L Quinidine). (**S**) Quantitative analysis of bone mineral density (BMD, mg/cm^3^) and bone volume fraction (BV/TV, %). Data are expressed as mean ± SD (*n* = 5 per group). Statistical significance was assessed using one-way analysis of variance with Holm–Šídák multiple-comparison tests: ns, not significant; * *p* < 0.05; ** *p* < 0.01; *** *p* < 0.001; and **** *p* < 0.0001.

To verify the osteoprotective effect of quinidine in a glucocorticoid-induced bone-loss model, we treated dexamethasone (Dex)-induced osteoporotic zebrafish with gradient concentrations of quinidine (1, 10 and 100 μmol/L). Alizarin Red staining revealed that quinidine effectively rescued Dex-impaired cranial bone mineralization. Compared with the Dex-induced model group, the 10 μmol/L quinidine group achieved the most significant improvements, with a 35.7% increase in cranial mineralization area and a 31.8% increase in intensity (*p* < 0.05). The 1 μmol/L group also showed clear beneficial effects, whereas the highest concentration (100 μmol/L) produced weaker or non-significant improvements. Consistently, transgenic Tg(*Ola.Sp7*:nlsGFP) zebrafish exhibited increased osteogenic fluorescent area and intensity after quinidine intervention, again with the 10 μmol/L group showing the optimal effect (Figure 3A–N). Notably, the concentration–response relationship was non-monotonic (biphasic) rather than classically dose-dependent: intermediate concentrations (especially 10 μmol/L) were more effective than the highest concentration tested. This pattern is pharmacologically plausible, as high concentrations of quinidine may introduce non-specific ion-channel blockade or cardiac-related toxicity that partially offsets its osteoprotective activity.

qRT-PCR analysis revealed the regulatory effects of quinidine on bone metabolism-related gene expression (Figure 3O). Low and medium concentrations of quinidine (1 and 10 μmol/L) significantly increased the expression of the osteoblast-associated gene col2a1a (*p* < 0.05), while the high-concentration group (100 μmol/L) showed no statistically significant upregulation. Genes involved in osteoclast activity and bone resorption (spp1 and ctsk) were markedly downregulated across all quinidine intervention groups (*p* < 0.05). These findings indicate that quinidine, at effective intermediate concentrations, modulates the expression of genes associated with osteoblast function and bone-resorption regulation in the dexamethasone-induced zebrafish model.

Micro-CT of adult zebrafish was further performed to evaluate bone structural changes (Figure 3P–S). Compared with the control group, the dexamethasone-treated group displayed obvious bone loss and structural deterioration in the skull and spine. Quinidine treatment markedly rescued these osteoporotic phenotypes and restored bone architecture. Quantitative analysis verified that quinidine significantly increased bone mineral density (BMD) and bone volume fraction (BV/TV), providing preliminary evidence of an association between quinidine treatment and improved bone parameters in adult zebrafish.

### 3.4. Quinidine Monotherapy Exerts Osteoprotective Effects

**Figure 4:**
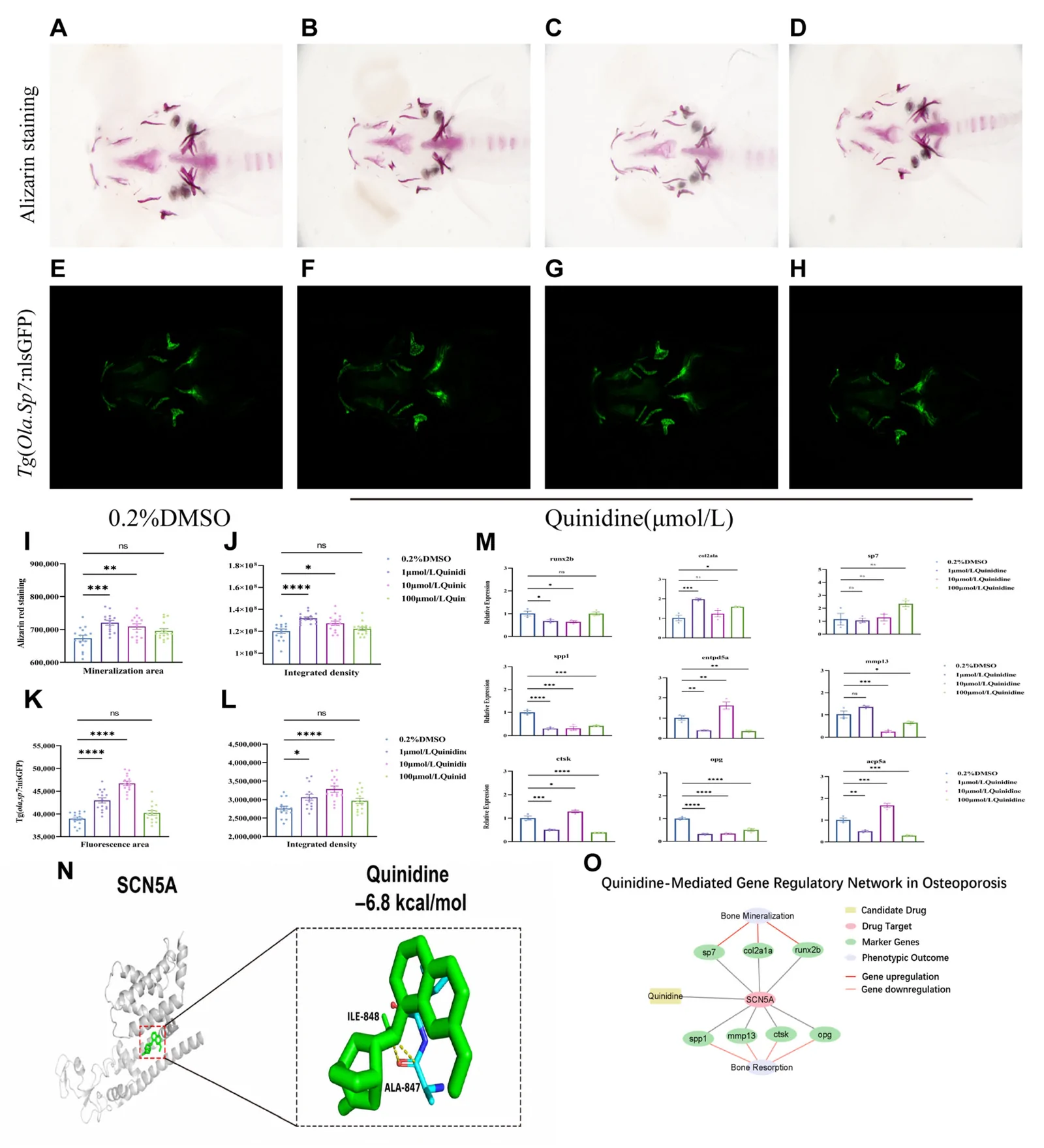
Effects of quinidine on bone-remodeling-related gene expression and the proposed regulatory mechanism. (**A**–**H**) Representative images of Alizarin Red staining of Tu zebrafish and fluorescence of Tg(*Ola.Sp7*:nlsGFP) after quinidine bath treatment alone. (**A**,**E**) 0.2% DMSO group; (**B**,**F**) 1 μmol/L quinidine group; (**C**,**G**) 10 μmol/L quinidine group; (**D**,**H**) 100 μmol/L quinidine group. (**I**–**L**) Quantitative results of the mineralized area and intensity of Alizarin Red staining and fluorescence after quinidine bath treatment alone. (**I**) Quantitative results of the mineralized area of Alizarin Red staining in Tu zebrafish; (**J**) Quantitative results of the mineralized intensity of Alizarin Red staining in Tu zebrafish; (**K**) Quantitative results of the fluorescent mineralized area of Tg(*Ola.Sp7*:nlsGFP) transgenic zebrafish; (**L**) Quantitative results of the fluorescent mineralized intensity Tg(*Ola.Sp7*:nlsGFP) transgenic zebrafish. (**M**) Results of qRT-PCR for marker genes of osteoblasts and osteoclasts after quinidine bath treatment alone. (**N**) Molecular docking analysis of Quinidine with the SCN5A protein. The binding energy is −6.8 kcal/mol, suggesting favorable predicted binding; this computational prediction has not been experimentally validated. (**O**) Hypothetical quinidine-mediated gene regulatory network in osteoporosis. Quinidine is hypothesized to modulate SCN5A/NaV1.5 channel activity and bone-remodeling-related gene expression; direct target-engagement, electrophysiological, and genetic validation was not performed. Data are presented as mean ± SD (n = 15 larvae per group for imaging outcomes; qRT-PCR comprised three biological replicates with approximately 50–60 larvae pooled per replicate). Statistical significance was assessed using one-way analysis of variance with Holm–Šídák multiple-comparison tests: ns, not significant; * *p* < 0.05; ** *p* < 0.01; *** *p* < 0.001; and **** *p* < 0.0001.

To further investigate the pharmacological activity of quinidine, we treated 3-day-old wild-type Tu and Tg(*Ola.Sp7*:nlsGFP) transgenic zebrafish with quinidine at gradient concentrations for five consecutive days. Alizarin Red staining and Image J quantitative analysis demonstrated that low and medium concentrations of quinidine exerted obvious osteogenic effects. In wild-type zebrafish, 1 μmol/L and 10 μmol/L quinidine significantly increased both cranial bone mineralization area and intensity (all *p* < 0.05), while 100 μmol/L quinidine produced no significant changes. Consistent results were observed in transgenic zebrafish: 1 μmol/L and 10 μmol/L quinidine markedly enhanced cranial osteogenic fluorescent area and intensity (*p* < 0.05), whereas 100 μmol/L quinidine showed no statistical difference (Figure 4A–L). Collectively, these results indicate that low and intermediate concentrations of quinidine were associated with increased cranial mineralization in zebrafish larvae.

qRT-PCR results showed that all three quinidine concentrations slightly increased the expression of osteoblast-associated genes (col2a1a and sp7) without reaching statistical significance (*p* > 0.05), but significantly downregulated spp1 and opg (*p* < 0.05) (Figure 4M). Note that opg is primarily produced by osteoblast-lineage cells and functions as a decoy receptor that inhibits RANKL-induced osteoclastogenesis; its downregulation therefore reflects altered osteoblast–osteoclast coupling signals rather than a direct measure of osteoclast activity. Collectively, these results indicate that quinidine altered the expression of several genes associated with bone remodeling. However, spp1 and opg are not specific markers of osteoclast activity. In particular, because OPG acts as a decoy receptor that inhibits RANKL-mediated osteoclastogenesis, the observed downregulation of opg should not be interpreted as evidence of suppressed osteoclast activity. Therefore, the transcriptional changes observed in this study reflect alterations in bone-remodeling-related gene expression but do not directly demonstrate inhibition of osteoclast formation or resorptive function. Direct measurements of osteoclast number and bone-resorption activity were not performed.

To explore a possible mechanistic link, molecular docking was performed and predicted a binding affinity of −6.8 kcal/mol between quinidine and the SCN5A protein (Figure 4N). Based on the computational drug-repositioning results, the MR association with SCN5A expression, and the observed changes in bone-related gene expression, we constructed a proposed SCN5A-centered regulatory network (Figure 4O). This network is presented as a working hypothesis only: it has not been validated by direct functional experiments such as SCN5A knockdown/knockout, electrophysiological recordings, or calcium-imaging assays in bone cells.

## 4. Discussion

This study integrated computational biology methods—including single-cell transcriptomics analysis, drug repositioning, Mendelian randomization, and a dexamethasone-induced zebrafish model—to identify and provide preliminary preclinical evidence for quinidine as a candidate osteoprotective agent warranting further investigation.

Firstly, through single-cell transcriptomics analysis, 16 cell types were successfully annotated, and 1647 differentially expressed genes (DEGs) in the osteoblast cluster were screened. GO enrichment analysis revealed that these genes are primarily involved in bone metabolism-related processes, such as extracellular matrix organization, ossification, cartilage development, and collagen metabolic process. Osteoblasts play a critical role in maintaining skeletal homeostasis, and their dysfunction is a central element in the development of osteoporosis [28]. Secondly, by employing the computational pipeline for drug repositioning based on single-cell data (ASGARD) [17], we identified four potential candidate drugs: perphenazine, imatinib, quinidine, and vinblastine. To prioritize among these candidates, we applied pre-defined selection criteria including statistical significance (FDR < 0.05), action on the osteoblast cluster, clinical accessibility, and limited prior investigation in the context of osteoporosis. Among these, perphenazine, imatinib, and vinblastine have been previously reported in relevant studies. Wang M et al. [29] found that the antipsychotic drug perphenazine can lead to reduced bone mineral density and increased incidence of osteoporosis, suggesting a need for caution regarding bone health risks. Imatinib, as a tyrosine kinase inhibitor, can decrease osteoclast number and activity, leading to bone metabolism disorders [30]. Ohya K et al. [31] experimentally demonstrated that vinblastine can cause hypocalcemia, possibly related to its interference with the regulatory mechanisms of calcium homeostasis in bone cells and its disruption of microtubules. Ross R et al. [32] also proved through relevant experiments that vinblastine can affect osteoblast function and the synthesis of bone matrix. Among the four candidates, perphenazine and imatinib—despite higher raw drug scores—were deprioritized because prior evidence indicates they adversely affect bone metabolism (perphenazine is associated with reduced bone mineral density and increased osteoporosis incidence; imatinib has been shown to reduce osteoclast number and activity, disrupting bone remodeling), making them mechanistically unfavorable as osteoprotective candidates. Quinidine, a Class Ia antiarrhythmic drug primarily used to treat Brugada syndrome and idiopathic ventricular fibrillation, had not previously been linked to osteoporosis research; combined with its favorable predicted drug-target profile, it was therefore selected for in-depth investigation and validation in this study.

Furthermore, the Mendelian Randomization (MR) analysis method is an important approach for inferring the causal relationship between an exposure (such as a biomarker, environmental factor, or drug target) and a disease [33]. The results showed a significant positive association between genetically predicted SCN5A expression and osteoporosis risk. Because quinidine is a known blocker of the NaV1.5 channel encoded by SCN5A, this genetic evidence provides supportive, though indirect and not definitive, rationale for further investigating quinidine’s osteoprotective potential. As noted in the Results, the MR analysis assessed the association between genetically predicted SCN5A expression and osteoporosis risk; it did not directly evaluate the pharmacological effect of quinidine. Moreover, although the zebrafish experiments assessed the effects of quinidine on bone-related phenotypes, they did not establish that these effects were mediated through SCN5A.

From a mechanistic perspective, quinidine is a Class Ia antiarrhythmic agent that primarily functions by inhibiting the voltage-gated sodium channel NaV1.5, which is encoded by the SCN5A gene. Although this channel is traditionally associated with cardiac electrophysiology, bioelectric signaling also contributes to skeletal homeostasis [34]. Voltage-gated sodium channels have been found to be functionally expressed in both osteoblasts and osteoclasts, where they regulate membrane potential, which in turn influences calcium signaling—a critical pathway for osteoblast differentiation and mineralization. By inhibiting sodium influx, quinidine may hyperpolarize the cell membrane or alter depolarization-dependent calcium entry, thereby promoting osteoblast activity (upregulation of runx2b and sp7) while suppressing osteoclastogenesis (downregulation of mmp13 and ctsk). This dual action restores bone remodeling balance [35]. Further mechanistic investigations, including SCN5A knockout models and electrophysiological studies in bone cells, are warranted to fully elucidate these pathways. Quinidine may therefore provide a link between ion-channel pharmacology and skeletal biology [36]. However, this mechanism remains hypothetical because SCN5A knockdown, channel-current recordings, and calcium-signaling assays were not performed in bone cells.

Finally, the experimental results from the zebrafish model further confirmed the pharmacological activity of quinidine. The zebrafish is an ideal model for osteoporosis research due to its convenient genetic manipulation, low rearing cost, short experimental cycle, and the many similarities between its skeletal development process and molecular regulation mechanisms and those in humans [37]. In this study, the osteoporosis model was established using 10 μmol/L Dex, which induces osteoporosis by inhibiting osteoblast differentiation and promoting osteoclastogenesis. Alizarin Red staining showed that Dex treatment significantly reduced the cranial bone mineralization area and intensity of the zebrafish. qRT-PCR confirmed the downregulation of osteoblast marker genes (*runx2b*, *col2a1a*, *sp7*) and the upregulation of osteoclast marker genes (*spp1*, *ctsk*), thus successfully establishing the osteoporosis model. The quinidine treatment results showed that, when combined with Dexamethasone, the 10 μmol/L Dex + 10 μmol/L Quinidine treatment group demonstrated the most pronounced improvement in cranial bone mineralization area and intensity, effectively alleviating the symptoms of osteoporosis.

Interestingly, we observed a non-monotonic (biphasic) concentration–response relationship, in which the intermediate concentration (10 μmol/L) produced the strongest osteoprotective effect, while the highest concentration (100 μmol/L) was less effective. This pattern is consistent with the known pharmacological profile of quinidine, a multi-channel blocker that can exhibit non-specific effects and potential toxicity at higher concentrations. Future studies should therefore carefully evaluate the therapeutic window and potential off-target effects of quinidine in bone tissue.

The main contribution of this study is the integration of single-cell transcriptomics-based drug repositioning, Mendelian randomization, and zebrafish experiments within an exploratory drug-repurposing framework. The MR analysis provided indirect genetic evidence concerning SCN5A expression and osteoporosis risk, while the zebrafish experiments provided preliminary in vivo evidence regarding the bone-related effects of quinidine; neither analysis established an SCN5A-dependent pharmacological mechanism. However, this study also has several limitations. First, the single-cell analysis is limited by the small number of biological replicates (only two osteoporotic fracture donors and three control donors). Treating individual cells as independent observations can lead to pseudoreplication and inflate statistical significance. Donor-level pseudobulk analysis confirmed this issue, as the number of significant differentially expressed genes was substantially reduced. Therefore, the osteoblast DEGs used for subsequent drug repositioning should be interpreted with caution, and future studies with larger donor cohorts are warranted. In addition, the ASGARD-based drug-screening analysis was performed using DEGs derived from the osteoblast cluster. Other cell populations, including immune, endothelial, and stromal cells, were not systematically incorporated into the drug-prioritization analysis. Therefore, the potential multicellular effects of quinidine within the bone microenvironment remain unclear. Moreover, because GSE217792 was derived from non-union fracture tissue, the observed transcriptional differences may partly reflect fracture-healing-related processes rather than systemic osteoporosis alone. Second, the integration of multi-omics data is insufficient; data such as proteomics and metabolomics were not included. Multi-omics integration could more comprehensively elucidate the mechanism of action of quinidine. Third, quinidine carries a known risk of cardiotoxicity. Further studies should carefully evaluate its therapeutic window, safety profile, and underlying mechanistic pathways. Fourth, this study relies exclusively on a zebrafish model and gene-expression-level readouts; direct functional measurements of osteoblast differentiation, osteoclast resorptive activity, dynamic bone formation, and bone mechanical strength were not performed, and no positive control using an established anti-osteoporosis agent was included. In addition, increased Alizarin Red staining and osteoblast marker expression in Dex-treated larvae primarily reflect effects on early skeletal development rather than adult bone remodeling or bone fragility; the extent to which these developmental findings translate to postnatal or age-related bone loss remains to be established, which is partly addressed by the complementary adult zebrafish Micro-CT experiments in this study. Furthermore, adult zebrafish in the dexamethasone plus quinidine treatment group showed sluggish swimming behavior, suggesting potential dose-related neuromuscular or cardiac effects consistent with quinidine’s known ion channel activity; systematic assessment of cardiac rhythm and other toxicity endpoints was not performed in this study and should be prioritized in future safety evaluations before considering quinidine’s therapeutic translation. Despite substantial skeletal developmental homology between zebrafish and mammals, cross-species differences in bone remodeling dynamics and drug metabolism mean that validation in mammalian osteoporosis models and primary human osteoblasts/osteoclasts will be necessary before the clinical relevance of these findings can be established. Fifth, the eQTLs used for SCN5A were derived from whole blood (eQTLGen Consortium) rather than bone tissue. An association based on non-skeletal eQTLs cannot directly establish a causal role for SCN5A specifically in osteoblasts or osteoclasts. Future studies using bone-specific eQTLs or functional experiments in bone cells are needed to confirm tissue-specific effects. Sixth, the concentration–response relationship in larval zebrafish was non-monotonic, with the highest concentration (100 μmol/L) generally less effective than 10 or 1 μmol/L. Larval survival, cardiac rhythm, developmental delay, and gross morphological abnormalities were not systematically quantified across concentrations. Given the known cardiotoxicity of quinidine, the possibility that toxicity contributed to the weaker response at 100 μmol/L cannot be excluded, and dedicated dose-optimization and safety studies are warranted. Seventh, the L1000 drug-response signatures used for the ASGARD-based drug repositioning were predominantly generated from cancer-derived cell lines under standardized in vitro drug concentrations. Their relevance to primary human osteoblasts, in vivo bone tissue drug exposure, and chronic osteoporosis treatment has not been directly demonstrated, and this should be considered when interpreting the drug prioritization results. In summary, this study systematically revealed the potential role of quinidine in the prevention and treatment of osteoporosis through single-cell drug repositioning, Mendelian Randomization, and zebrafish experimental validation. Quinidine treatment was associated with the promotion of bone mineralization and increased bone mineral density in the zebrafish model, providing a preliminary perspective for drug repositioning research in osteoporosis that warrants further mechanistic and statistical validation.

## 5. Conclusions

In this study, an integrated strategy combining single-cell transcriptomics-based drug repositioning, Mendelian randomization, and zebrafish model validation identified quinidine as a promising candidate for further investigation as a potential osteoprotective agent in the context of glucocorticoid-induced bone loss. The results demonstrate the feasibility of this multi-layered computational–experimental pipeline for osteoporosis drug discovery. However, given the preliminary nature of the zebrafish model, the small effect size observed in the MR analysis, and the as-yet-untested SCN5A mechanism, quinidine should currently be regarded as a hypothesis-generating candidate requiring mechanistic validation, safety/pharmacokinetic assessment, and testing in mammalian models before its therapeutic relevance for osteoporosis can be established. This integrated framework nonetheless offers a useful methodological reference for future anti-osteoporosis drug repositioning studies.

## Figures and Tables

**Table 1:** Raw Therapeutic Scores of Four Candidate Drugs for the Osteoblast Cluster.

	Drug Therapeutic Score	*p*-Value	FDR	Cell Type
perphenazine	1.36	4.68 × 10^−7^	3.69 × 10^−4^	Osteoblast
imatinib	0.72	1.80 × 10^−5^	1.80 × 10^−2^	Osteoblast
quinidine	0.66	9.11 × 10^−5^	2.54 × 10^−2^	Osteoblast
vinblastine	0.50	7.99 × 10^−5^	2.54 × 10^−2^	Osteoblast

## Data Availability

The original contributions presented in this study are included in the article/Supplementary Material. Further inquiries can be directed to the corresponding author(s).

## Supplementary Materials

The following supporting information can be downloaded at: https://www.mdpi.com/article/10.3390/genes17091060/s1, Figure S1: Donor contribution across cell clusters; Figure S2: Leave-one-out sensitivity analysis for the Mendelian randomization association between SCN5A expression and osteoporosis risk; Table S1: Donor-level pseudobulk differential expression results of osteoblasts (DESeq2); Table S2: F-statistics of the instrumental variables for SCN5A.
