# Supplementary material for: Drug Repositioning Based on Single-Cell Transcriptomics Data Identifies Quinidine as a Potential Drug for Treating Osteoporosis
Source: Genes (Basel). 2026 Aug 31;17(9):1060. doi: 10.3390/genes17091060 (PMC13606602; doi:10.3390/genes17091060)
Supplement: Supplementary file 1 [file genes-17-01060-s001.zip › genes-4455273-supplementary.pdf]

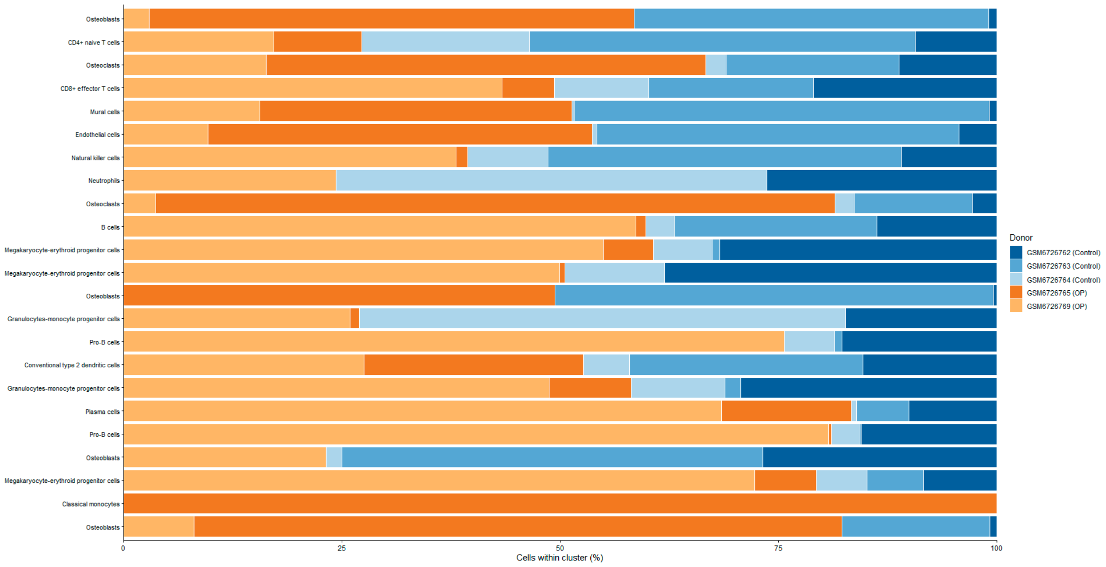

Supplementary Figure S1. Donor contribution across cell clusters. Stacked bar plot showing the proportion of cells from each donor (Sample_ID) within each annotated cell cluster. GSM6726762, GSM6726763 and GSM6726764 are control donors; GSM6726765 and GSM6726769 are osteoporotic fracture donors. Major clusters of interest, including osteoblasts, contain cells from multiple donors and are not dominated by a single sample.

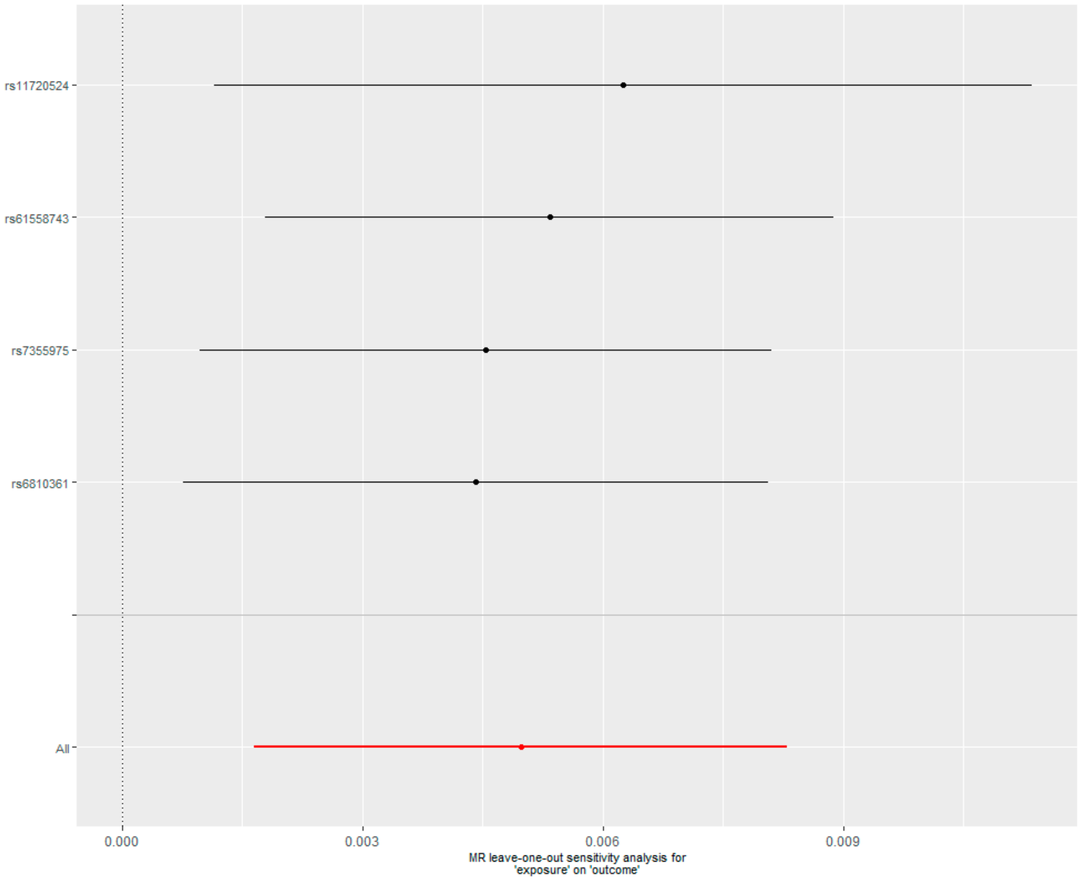

Supplementary Figure S2. Leave-one-out sensitivity analysis for the MR association between SCN5A expression and osteoporosis risk. Each black point represents the inverse-variance weighted (IVW) causal estimate after excluding the indicated SNP. The red point represents the overall IVW estimate using all four instrumental variables. Horizontal lines indicate 95% confidence intervals. The overall estimate remained stable and the 95% confidence interval excluded the null across all leave-one-out iterations, indicating that no single SNP disproportionately drove the association.

Supplementary Table S1.Donor-level pseudobulk differential expression results of osteoblasts (DESeq2)

Gene	log2FoldChange	padj
APOL4	-4.34	4.99×10^{-4}
CD36	-4.16	0.014
ACAN	4.46	0.033
IFITM1	-2.51	0.033
AL109811.3	-6.79	0.038

Supplementary Table S2. F-statistics of the instrumental variables for SCN5A.

SNP	β	se	F-statistic	SNP
rs11720524	-0.121	0.0124	95.45	rs11720524
rs6810361	0.080	0.0148	28.92	rs6810361
rs7355975	-0.099	0.0211	22.01	rs7355975
rs61558743	-0.066	0.0143	21.48	rs61558743
Mean	-	-	41.96	
